# Influence of Brake Pad Temperature Variation on the Squeal Noise Characteristics of Disc’s In-Plane Vibration Mode

**DOI:** 10.3390/s25134080

**Published:** 2025-06-30

**Authors:** Sungyuk Kim, Seongjoo Lee, Shinwook Kim, Jaehyeon Nam

**Affiliations:** 1 Technical Center, KB Autosys Co., Ltd., Asan-si 31443, Republic of Korea; sykim5641@naver.com (S.K.); sjlee@kbautosys.com (S.L.); swkim@kbautosys.com (S.K.); 2 Mechanical Convergence System Center, Institute for Advanced Engineering, Youngin-si 17180, Republic of Korea

**Keywords:** mode coupling, squeal noise, in-plane mode, thermal expansion, brake dynamometer

## Abstract

This study investigated the squeal noise characteristics of the in-plane mode of the disc in a disc brake system as influenced by the temperature of the brake pad. The temperature range of the brake pad was set between 50 °C and 300 °C, and the squeal noise was analyzed by calculating the complex eigenvalues using the finite element method (FEM). The FEM analysis indicated that instability was most sensitive near 80 °C, and it was observed that instability exhibited mode exchange from the disc’s in-plane mode to the out-of-plane mode in a nearby frequency band due to thermal deformation of the pad. A reproduction test was conducted using a brake dynamometer, where the main squeal noise was found to be approximately 10,000 Hz, consistent with the FEM analysis. Additionally, the squeal noise occurred most near 100 °C, and the noise disappeared after 250 °C. These results largely align with the FEM analysis model, validating the suitability of the analysis approach.

## 1. Introduction

Frictional vibration is a significant issue for various systems subjected to friction, such as doors, brakes, and seats. The unstable vibration caused by friction leads to noise, thereby causing emotional quality problems. Considering noise is directly related to the stability of a system, its root cause should be analyzed. The vibration caused by friction in a brake is typically classified into groan noise, which occurs near a low frequency of 500 Hz, and squeal noise, which occurs at a higher frequency [1,2,3]. Considering that automotive brakes are directly related to safety, relatively unstable vibration is the main cause of many customer complaints and overall automotive quality issues.

Research on brake noise has been conducted using various methods, including experimental methods using dynamometers, theoretical verification methods for obtaining complete solutions, and complex eigenvalue analysis based on the finite element method (FEM). Because squeal noise is the most sensitive in the audible frequency range (20 Hz–20 kHz), many studies have been conducted to identify its causes [4]. Brake squeal is well known as frictional self-excited vibration, and many studies have applied theoretical approaches to investigate the dynamic instability of brake systems [5,6,7]. Mode-coupling instability, known as the main cause of brake squeal, can be described using a spring-mass model. In this model, mode-coupling instability is caused by frictional coupling in the critical region [8,9]. Mathematical approaches can directly express the mechanism of mode-coupling instability based on complete solutions; however, reflecting geometric elements is difficult. Sinou et al. [10] derived characteristic equations by the linearization of nonlinear systems and analyzed the stability of friction systems using stability theory. The stability analysis was conducted based on complex eigenvalue analysis and the Routh–Hurwitz criterion, and mode-coupling instability for damping elements was analyzed. Kang [11] identified the relationship between mode-merged vibration and the stick-slip limit cycle by building single-degree-of-freedom and coupled oscillator models. Another study by the author conducted a complex stability analysis that included gyroscopic, negative slope, and mode-coupling mechanisms for understanding the dynamic instability of disc brakes [12].

Improved mathematical approaches may reflect some geometric elements by expressing the equations of motion at contact points using a semi-analytical model; however, accurately expressing the equations of motion is extremely difficult and time-consuming. Nam et al. [13] investigated the instability caused by the negative slope and mode-coupling mechanisms by describing the equations of motion for a pin-on-disc at the contact points. The equations of motion could be expressed relatively simply as they were described at the contact points, and they could be rapidly implemented because the conditions for dynamic instability were clear. The rail pad model examined the fundamental squeal mode of dynamic instability occurring in the brake pad using refined equations of motion [14]. Kang et al. [15] analyzed the effect of dynamic instability on the contact span by representing physical models based on the vibration modes of the finite element models of a disc and a pad.

Recently, machine learning has been applied to various fields, including the classification and prediction of vibration signals [16]. Signals generated by dynamic instability can be visualized using a range of signal-processing techniques and subsequently classified using algorithms that analyze visual patterns, such as convolutional neural networks (CNNs) [17]. Since machine learning relies on data rather than analytical models, it does not require complex equations of motion or detailed physical modeling. However, its practical application remains challenging, as reliable estimation and classification demand large volumes of squeal data.

FEM-based analytical methods can predict the dynamic instability caused by friction based on algorithms defined in commercial software programs and models. Previously, their use was difficult owing to the considerable computation time and cost required; however, advances in hardware and software have made these methods relatively easily accessible. Ouyang et al. [18] introduced transient and complex eigenvalue analyses as numerical approaches for obtaining disc brake squeal and described the squeal mechanism and various parameters. Massi et al. [19] identified the cause and mechanism of the noise generated in thin plates by linear and nonlinear numerical approaches. Obert et al. [20] predicted squeal in the time domain by representing nonlinear models as finite element models to improve the reliability of existing linear stability analysis. The nonlinear time-domain analysis revealed instability even in the pad mode, which was not found by the existing mode-coupling instability analysis, and emphasized instability for the intermittency route type.

In recent years, various approaches have been adopted for studying brake squeal. By integrating various parameters and mechanisms, research can effectively simulate practical phenomena and elucidate distinct characteristics through traditional methodologies. Meehan et al. [21] analyzed the causes of brake noise by integrating various mechanisms that had been studied individually into a single model. The proposed model was used for a closed-form analysis of brake squeal occurrence. They also analyzed squeal characteristics in the time domain. Qi et al. [22] analyzed the mechanism of brake squeal that occurs in railway vehicles and its influencing factors, and defined squeal parameters by nonlinear analysis. They defined the brake pressure as the most important cause of brake noise, and based on experimental and analysis results, verified that the probability of brake squeal further increases with an increase in brake pressure.

Brake squeal, which results from relative motion caused by friction, is generated by self-excited vibration. The main factors of dynamic instability are known to be pressure, variations in friction coefficient, contact area, and damping—all of which are directly related to friction force. Although the stiffness and nonlinear stiffness behavior of the brake pad are important factors in determining dynamic instability, many classical studies have investigated these aspects through contact stiffness [11,12,15]. While contact stiffness is a critical factor influencing squeal in brake systems, it is difficult to define physically, and in actual brake systems, squeal frequently occurs due to physical factors other than the main parameters of friction force.

In complex eigenvalue analysis, when the friction coefficient is defined as a function of velocity, an increase in velocity coupled with a decrease in the friction coefficient leads to an increase in the positive real part of the eigenvalue, indicating enhanced instability. Temperature variations affect both velocity and the friction coefficient, leading to the development of corresponding dynamic instability conditions. Mortazavi et al. [23] theoretically investigated the conditions under which frictional sliding becomes unstable when the coefficient of friction varies with temperature. They demonstrated that friction generates heat, and if the coefficient of friction increases with temperature, a positive feedback loop is formed between heat generation and friction, potentially leading to system instability. Xiao et al. [24] analyzed the friction and wear characteristics of brake materials. Their study reported that during the initial phase of braking, the coefficient of friction may remain stable or slightly increase with rising temperature; however, once a certain threshold temperature is exceeded, significant degradation of the friction coefficient occurs due to physical and chemical changes at the contact surface. Balotin et al. [25] conducted an analysis that considered the temperature dependency of the coefficient of friction. They described that frictional heating is strongly influenced by material properties, and materials whose friction coefficients increase with temperature tend to generate more frictional heat. They also predicted that assuming a constant friction coefficient in FEM simulations can result in the underestimation of brake temperature. Although various studies have addressed the thermal and frictional behavior of brake systems, the trends vary significantly depending on the material and operating conditions, making it challenging to generalize a model.

Therefore, this study defines the contact characteristics according to temperature variations of the pad in a disc brake system and aims to investigate the characteristics of squeal noise in the in-plane mode, which is the main instability mode triggered by temperature change. Brake squeal research has primarily focused on the out-of-plane modes (doublet modes) of the disc. However, in the experimental results of the present study, dynamic instability did not occur in the out-of-plane mode of the disc. To evaluate instability modes, complex eigenvalue analysis using the finite element method (FEM) was conducted to represent all geometric characteristics. The pad temperature was set from 50 °C to 300 °C, and the corresponding instability modes were analyzed. Finally, brake dynamometer tests were conducted to verify the reproducibility of squeal noise in the disc’s in-plane mode, and the squeal characteristics were analyzed according to the pad geometry and temperature to validate the suitability of the FEM model.

## 2. Method

### 2.1. Complex Eigenvalue Analysis Using FEM

**Figure 1:**
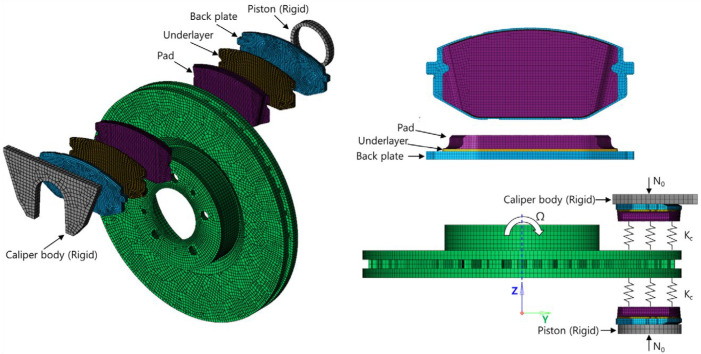
FE model of the brake system.

As shown in Figure 1, the finite element model of the disc brake system of a mid-size van is composed of a disc, a pad, an underlayer, and a backplate. The piston and caliper body, which transmit pressure to the pad, are simplified as rigid bodies. The pseudo-rotation [13] state, in which the disc rotates at a constant speed (Ω) with respect to the z-axis, is assumed. The pad is subjected to pressure under a constant load (N0) by the piston, resulting in a friction force that is caused by the relative motion between the disc and the pad on the contact surface. The contact pressure can be expressed by the static pressure (N0) and contact stiffness. For contact coupling, the contact stiffness between the disc and the pad, kc, was modeled using the penalty method. A caliper applies pressure with one piston, and the surface symmetrical to the piston is the caliper body. In this study, complex eigenvalue analysis was conducted using ABAQUS/Standard 2024. This method provides solutions for the real and imaginary parts of the eigenvalues and the damping ratio. The simulation model consists of a total of 112,776 hexahedral elements, and the material properties are summarized in Table 1. Since this study focuses on squeal instability due to temperature variation in the brake pad, the stiffness of the friction material was assumed to be linear.

The equation of motion of the brake system is as follows:

(1) $$\left[M\right]\ddot{u}+\left[C\right]\dot{u}+\left[K\right]u=0$$

where [M] is the mass matrix, [C] is the damping matrix, [K] is the stiffness matrix, and u is a displacement vector.

The characteristic equation can be written as:

(2) $$\left(\lambda^{2}\left[M\right]+\lambda \left[C\right]+\left[K\right]\right)\Phi =0$$

where λ denotes the eigenvalue and Φ represents the corresponding eigenvector. According to the definition of modal analysis, the damping matrix is neglected, and the stiffness matrix is assumed to be symmetric. For such symmetric systems, the resulting eigenvalues are purely imaginary and can be expressed as λ=iω. Thus, the undamped eigenvalue problem is given by:

(3) $$\left(\lambda^{2}\left[M\right]+\left[K_{s}\right]\right)\Phi =0$$

where $\left[K_{s}\right]$ is the symmetric stiffness matrix and ω is the circular frequency. To analyze the stability of the system due to friction, the eigenvectors obtained from the modal analysis are projected onto a reduced subspace, leading to the following complex eigenvalue problem:

(4) $$\left(\lambda^{2}[M^{*}]+\lambda [C^{*}]+[K^{*}]\right)\Phi^{*}=0$$

The projected stiffness matrix $\left[K^{*}\right]$ can be expressed as:

(5) $$\left[K^{*}]={\left[\phi_{1},\ldots ,\phi_{N}\right]}^{T}\left[K\right][\phi_{1},\ldots ,\phi_{N}\right]$$

(6) $$\left[K^{*}]=[K_{s}]+[K_{asym}\right]$$

Here, $\left[K_{asym}\right]$ is the friction-coupling matrix, which includes the effects of contact stiffness and friction forces. This asymmetric component of the stiffness matrix is responsible for inducing dynamic instabilities, such as squeal.

The complex eigenvalue problem is solved using the QZ method, and the eigenvectors of the original system are reconstructed as:

(7) $$\Phi =[\phi_{1},...,\phi_{N}]\Phi^{*}$$

The eigenvalue λ is a complex number consisting of a real and an imaginary part, as shown below:

(8) λi 1,2=Reλi+iIm(λi)

The real part of the eigenvalue determines the dynamic stability of the system. A positive real part indicates dynamic instability, which is characteristic of brake squeal phenomena.

In the complex eigenvalue analysis, the mounting holes for fastening the disc to the hub with bolts were fully constrained. For the pad, a tie condition (full coupling) was applied to the contact surfaces between the pad, underlayer, and backplate to prevent any relative displacement or rotation among the components. A pressure of 20 MPa was applied to the inner back plate using a piston to represent the pressure acting on the inner pad. For the outer pad, the caliper body was fully constrained to allow pressure transmission. The rotational speed of the disc was set to 2.49 rad/s, corresponding to a vehicle speed of approximately 3 km/h. As mentioned earlier, disc rotation in complex eigenvalue analysis is defined as pseudo-rotation, and the applied pressure—when not considering velocity-dependent terms—affects only the equilibrium state, assuming constant contact stiffness. Therefore, the applied pressure was defined as the maximum pressure that may occur in a typical automotive brake system.

### 2.2. Material Properties

**Figure 2:**
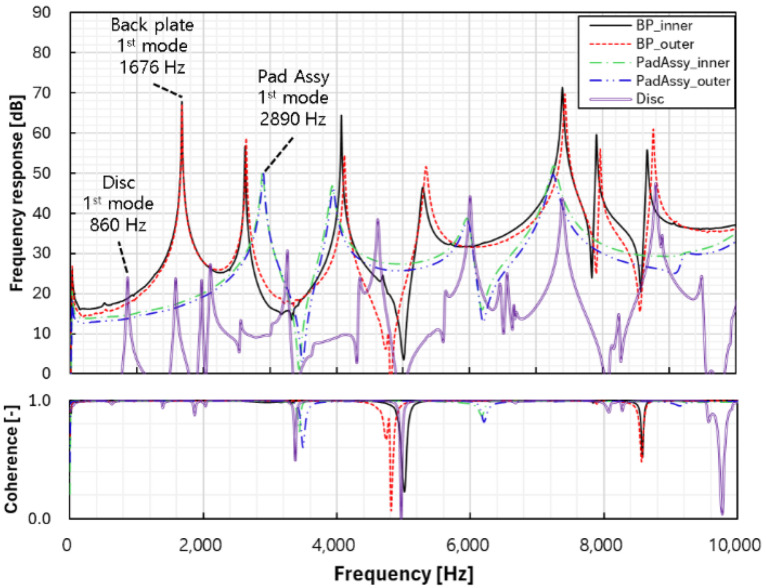
Modal test results for each brake component.

For the pad used in this study, materials with an iron content of 50% or higher, which are classified as semi-metallic, were used. The disc was made of gray cast iron and was similar in size to the discs of typical passenger cars. The physical properties of the disc and pad were corrected based on the modal test results after conducting FEM modal analysis using the elastic modulus, Poisson’s ratio, and density measured in advance. To implement the free-free boundary condition, a flexible elastic sound-absorbing material was used in the modal test, and the frequency response of each component was measured using an impact hammer based on the impact excitation technique. The impact was applied to the backplate, which has a dominant influence on the bending mode of the pad. The modal test was conducted using a uniaxial accelerometer, and the average value was obtained from five repeated measurements. The natural frequencies were validated by coherence, and the mode shapes were estimated by numerical analysis. The accelerometer was attached to the end of the backplate, where a large displacement was expected, based on the prediction of the first bending mode. Figure 2 shows the frequency response curve measured in the modal test. The primary and secondary natural frequencies (bending and torsional modes, respectively) identified from the backplate and pad assembly were used as factors to correct the elastic modulus and Poisson’s ratio, respectively. For the disc, the mode shape was the second nodal diametrical mode for the primary natural frequency and the nodal circle axial mode for the secondary natural frequency. The elastic modulus and Poisson’s ratio were corrected using the primary and secondary modes. Table 2, Table 3 and Table 4 compare the FEM analysis and test results for the natural frequencies of each component. The error rate for up to the tertiary mode was found to be less than 5%.

Contact stiffness, i.e., the change in contact pressure per unit length (as expressed in (9)), was estimated from the elastic modulus corrected from the modal test. The calculated contact stiffness of the pad was $1.45\times 10^{12}\;N/m^{3}$, and complex eigenvalue analysis was conducted by applying this value as a contact stiffness factor in the finite element analysis as follows:

(9) $$k_{c}=P_{c,\;max}/u_{max}\;[N/m^{3}]$$

where $P_{c,max}$ is the maximum contact pressure and $u_{max}$ is the maximum deformation.

**Figure 3:**
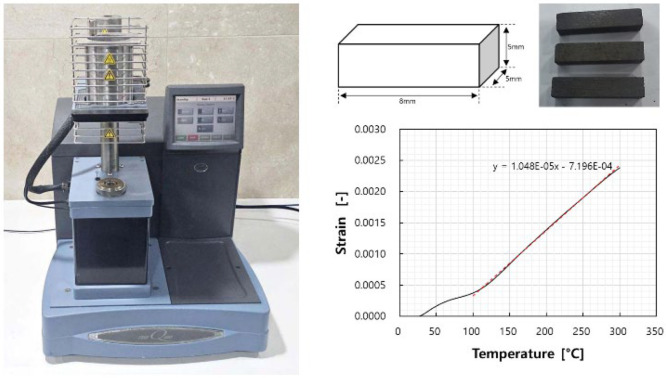
Measurement of the coefficient of linear expansion using a thermomechanical analyzer.

To reflect the thermal deformation characteristics of the brake pad, the coefficient of thermal expansion is measured by collecting specimens, as shown in Figure 3. Thermal expansion represents the change in the volume of the material due to the temperature change. As expressed in (10), the thermal strain, $\varepsilon_{T}$, is proportional to the temperature as follows:

(10) $$\varepsilon_{T}=\alpha \Delta T$$

where $\varepsilon_{T}$ is the thermal strain [−], α is the coefficient of linear expansion [/°C], and ΔT is the temperature change [°C].

In this study, the coefficient of linear expansion was used to reflect the thermal deformation characteristics. In the test, the coefficient of linear expansion was measured using a thermomechanical analyzer (TMA Q400 model, TA Instruments, New Castle, DE, USA). For the test, three specimens were precisely cut from one brake pad by selecting the *x*-axis direction as the longitudinal direction. Their sizes were set as 5 mm (width) × 5 mm (height) × 8 mm (length). The initial load was 0.1 N, and the temperature was increased from 27 °C to 300 °C at a rate of 5 °C/min. The thermal deformation of the pad according to the temperature was found to exhibit strong linearity after 100 °C, and the coefficient of linear expansion was calculated for the straight slope in the range of 100 to 300 °C. The coefficient of linear expansion of the pad was found to be $1.048\times 10^{-5}$/°C. For the disc, the coefficient of linear expansion of typical cast iron ($1.3\times 10^{-5}$/°C) was selected and used in the finite element analysis.

## 3. Results and Discussion

### 3.1. Complex Eigenvalue Analysis with Pad Temperature Variation

In this study, squeal noise that occurs in the in-plane mode was predicted using complex eigenvalue analysis [26,27]. In addition, an analysis was conducted by adding a thermal deformation phase to investigate the influence of thermal deformation. To this end, first, complex eigenvalue analysis according to the change in the friction coefficient was conducted without the application of thermal deformation. The in-plane mode was defined as the disc’s in-plane radial mode, characterized by dominant deformation in the radial direction. The out-of-plane mode was defined as a transverse doublet mode of the disc with *n* nodal diameters.

**Figure 4:**
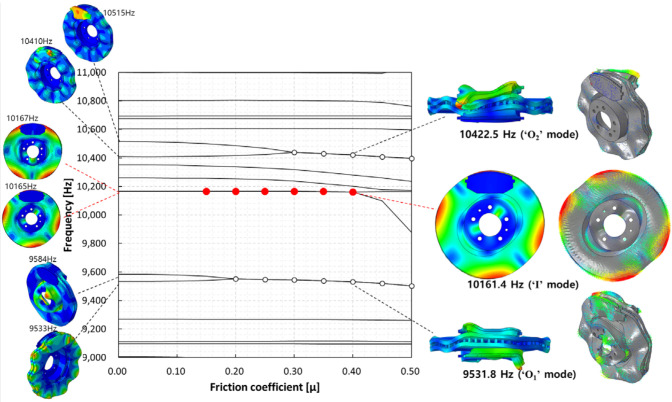
Complex eigenvalue analysis results without temperature conditions.

The results are shown in Figure 4. The main focus of this study was the in-plane mode of the disc, and the in-plane mode caused by the instability mode was identified at 10,161.4 Hz. Based on this, two dynamic instabilities were observed, for which the out-of-plane modes of the pad and disc were found to be the dominant modes at 9531.8 and 10,442.5 Hz. To distinguish, the instability modes at 9531.8 Hz, 10,442.5 Hz, and 10,161.4 Hz are referred to as “O1,” “O2,” and “I,” respectively. The “I” mode, identified as the instability mode caused by mode coupling between the two adjacent second in-plane compression modes, occurred at a friction coefficient of 0.15–0.4. The “O1” mode, developed at a friction coefficient of 0.2–0.5, was found to be the dynamic instability caused by mode coupling between the nodal diametrical mode of the disc and the bending mode of the pad (9533 Hz). Finally, the “O2” mode, developed at a friction coefficient of 0.3–0.5, was identified as mode coupling between the torsional mode of the pad and the high-order bending mode of the (10,410 Hz) disc. These results indicate that the developed brake system exhibits mode-coupling instability caused by mode-merging across various vibration modes. This study defined the dynamic instability caused by the in-plane mode as the main target and examined its characteristics.

For dynamic instability, friction coupling and characteristics that occur on the contact surface are dominant. As previously discussed, nonlinear stiffness and contact area are critical factors influencing dynamic instability, as they alter friction coupling and contact characteristics. In complex eigenvalue analysis, these effects are reflected in the modeling of the equilibrium state. In the present study, the equilibrium condition was represented by thermal deformation, in addition to the inherent material nonlinearity, and dynamic instability was investigated based on this modeling approach.

**Figure 5:**
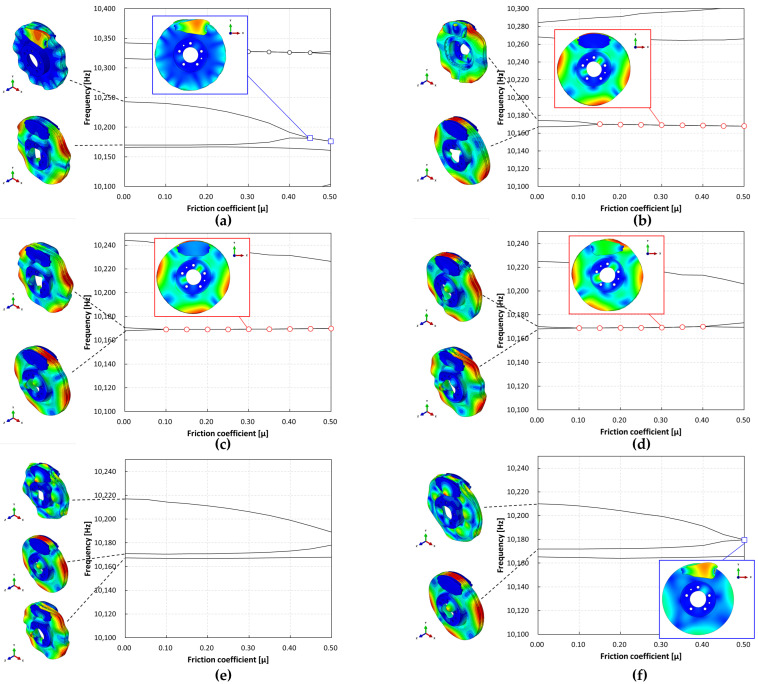
Complex eigenvalue analysis results by temperature. (**a**) 50 °C, (**b**) 100 °C, (**c**) 150 °C, (**d**) 200 °C, (**e**) 250 °C, (**f**) 300 °C.

The characteristics of the contact surface are deformed by frictional heat, which also leads to mode-coupling instability owing to the change in the equilibrium caused by the contact pressure. Therefore, complex eigenvalue analysis was conducted by applying thermal deformation according to the temperature conditions of the pad surface. Figure 5 shows the results of the analysis by designating the friction coefficient as a parameter for the model, applying thermal deformation at 50 °C intervals from 50 to 300 °C. At 50 °C, mode coupling developed between the in-plane mode of the disc and the *x*-axis direction rigid body mode of the pad, and the geometry of the instability mode was dominated by the deformation of the pad. At 100 °C, mode coupling occurred between the two closed disc in-plane modes and persisted across a friction coefficient range of 0.15–0.5. The same phenomenon also occurred at 150 °C, and the instability mode occurred continuously for a friction coefficient between 0.1 and 0.5. The same result also occurred at 200 °C, and the instability mode occurred for a friction coefficient between 0.05 and 0.4. The instability mode did not develop at 250 °C. Finally, at 300 °C, the instability mode geometry dominated by the in-plane mode of the disc and the *x*-axis rigid body mode of the pad occurred at a friction coefficient of 0.5. The characteristics of the pure in-plane mode of the disc were independent of changes in the contact stiffness. However, the “I mode” was partially affected by the out-of-plane mode in the *z*-axis direction rather than the in-plane mode. Therefore, the deformation in the *z*-axis direction by the head of the pad indicates that the mode coupling will be changed from (in-plane + in-plane) to (in-plane + out-of-plane).

**Figure 6:**
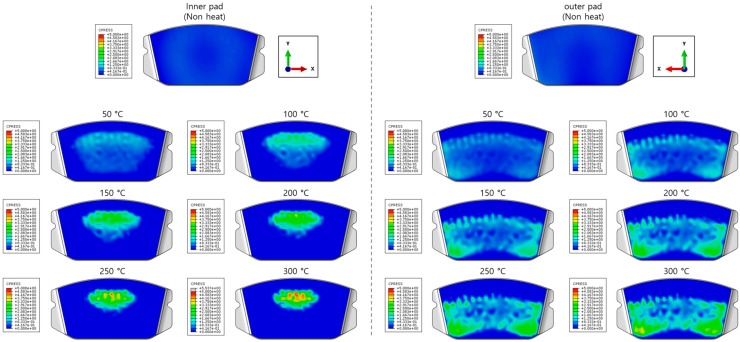
Pressure distribution of the pad due to thermal deformation.

Figure 6 shows the change in the pressure distribution due to the thermal deformation caused by the temperature rise of the pad. The contact pressure of the inner pad was caused by the piston load, and the pressure distribution was in response to the initial geometry of the piston. Thermal expansion transformed the pad assembly into a bow shape, thereby resulting in pressure concentration at the center. The outer pad corresponded to the caliper body. Considering it was designed to support the edge of the outer backplate rather than its center, the pressure was concentrated on the edge. Therefore, deformation of the inner and outer pad assemblies occurred in an asymmetric structure, and the difference between the contact and friction forces of the disc was determined as the main characteristic. Therefore, the change in the contact pressure caused by the temperature change leads to a change in the main mode that develops mode coupling, thereby indicating that it is a major factor for squeal sensitivity.

**Figure 7:**
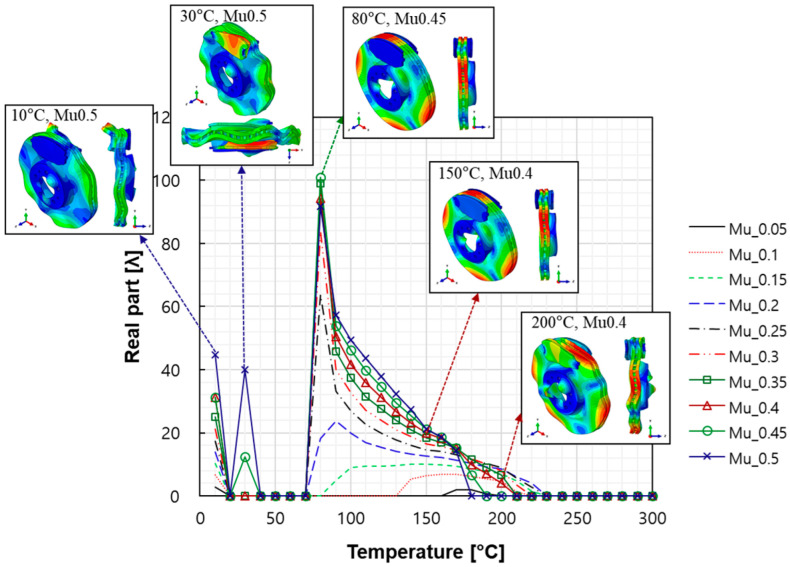
Real part variation in disc in-plane mode with temperature and friction coefficient changes.

Figure 7 shows the magnitude of the real part in the in-plane mode according to the temperature and friction coefficient. The magnitude of the real part was largest near 80 °C, thereby indicating that the instability caused by thermal deformation was the most sensitive in this region. The real part was zero after 230 °C, thereby showing that the instability of the in-plane mode disappeared in the region.

The squeal sensitivity due to temperature variation is attributed to thermal deformation, which alters the pad’s contact area, as shown in Figure 6. This contact area is regarded as a major factor in squeal sensitivity associated with mode-coupling instability, where dynamic instability tends to occur in specific contact regions [12,27,28]. Therefore, the occurrence of mode-coupling instability within certain temperature ranges suggests that thermally induced changes in contact stiffness and geometry contribute to the tendency for squeal generation.

When the mode shape was examined, the in-plane and out-of-plane modes (pad, disc) were mixed below 80 °C. The contribution of the in-plane mode tended to increase in the 80–180 °C region, and that of the out-of-plane mode tended to increase above 200 °C. Specifically, the contribution of the out-of-plane mode to instability increased according to the *z*-axis deformation of the pad. This indicated that the instability mode changed into the out-of-plane mode by the deformation of the pad caused by temperature for squeal noise, which was dominated by the in-plane mode of the disc. As explained above, the instability mode occurring in the in-plane mode is unrelated to contact stiffness. However, the thermal deformation of the pad caused a change in the mode coupling from the out-of-plane mode in the nearby frequency range. Therefore, although the main cause of squeal noise by the in-plane mode is the disc, both the disc and the pad need to be improved owing to the complex instability caused by the thermal deformation of the pad.

### 3.2. Brake Dynamometer Test Verification

**Figure 8:**
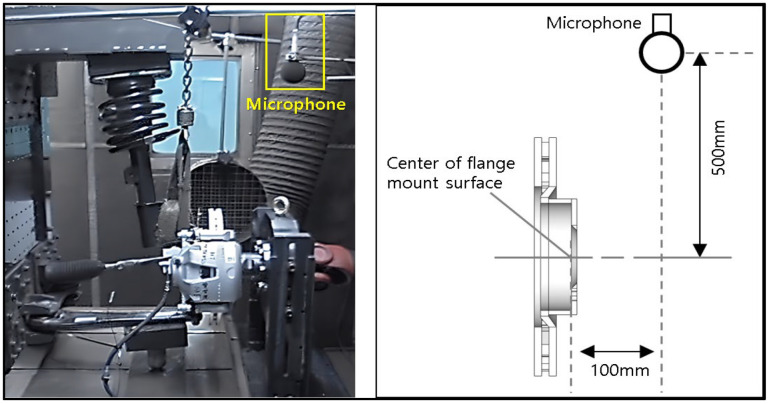
Brake dynamometer test set up and test samples.

A brake dynamometer test was conducted in accordance with SAE J2521 using equipment from Link Engineering Company (Detroit, Michigan, USA) to verify the squeal characteristics of the disc in-plane mode derived from finite element analysis. SAE J2521 (disc and drum brake dynamometer squeal noise test procedure) presents a common method for performing a series of selected test sequences to identify whether a brake assembly will develop squeal noise under various test conditions, as shown in Figure 8. SAE J2521 presents a method for identifying the occurrence of squeal noise in the brake system under various test conditions. The test consists of a total of 31 modules and can be broadly divided into four sections as follows:

A. Bedding section (Modules 1–3): A burnishing phase for the initial stabilization of the disc and pad.

B. Warm drag section (Modules 4–18): A phase for evaluating drag performance under normal temperature conditions.

C. Cold drag section (Modules 19–24): A phase for evaluating drag performance under low temperature conditions.

D. Recovery section (Modules 25–31): A phase for evaluating the recovery of friction performance after the fade test.

In this study, to minimize the effect of wear, the test was conducted using Modules 1 to 8, which include the Bedding section and a portion of the Warm drag section. A summary of the test modes used is provided in Table 5.

**Figure 9:**
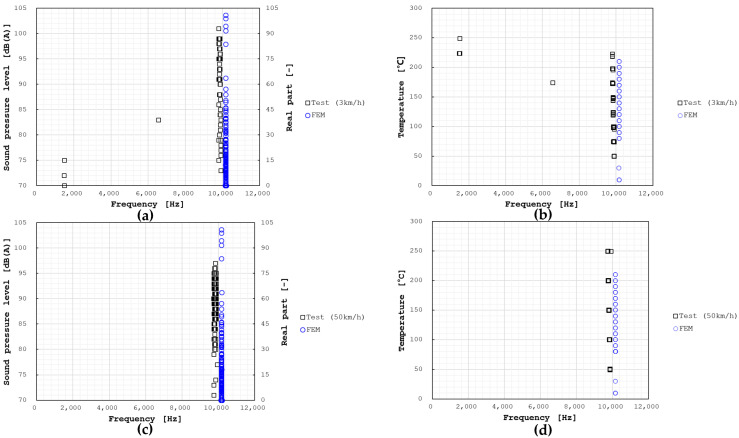
Squeal noise by brake dynamometer test and FEM results. (**a**) Frequency vs. Sound pressure level (at 3 km/h), (**b**) Frequency vs. Temperature (at 3 km/h), (**c**) Frequency vs. Sound pressure level (at 50 km/h), (**d**) Frequency vs. Temperature (at 50 km/h).

**Figure 10:**
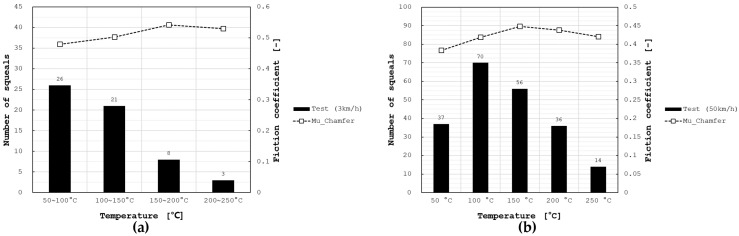
Number of squeal noises by brake pad temperature. (**a**) 3 km/h, (**b**) 50 km/h.

Figure 9 illustrates the squeal noise results measured in the SAE J2521 test according to sound pressure level and temperature. Squeal noise predominantly occurred at speeds of 3 km/h and 50 km/h, and consistent with the simulation results, the noise near 10,000 Hz was dominant throughout the entire test section. Intermittent squeal noise was also observed at 1450 Hz, 2750 Hz, 3400 Hz, 4300 Hz, and 6550 Hz; however, the main squeal events occurred around 10,000 Hz. Squeal events according to temperature are shown in Figure 10. At a speed of 3 km/h, squeal events tended to decrease as the temperature increased. At 50 km/h, the greatest number of squeal events occurred at 100 °C, after which they decreased and disappeared completely at 300 °C. This trend closely matched the change in the real part with temperature, thereby confirming the reliability of the analysis results. The friction coefficient mostly showed an increasing trend with rising temperature, which implies that the test was conducted within a temperature range where decomposition of the thermosetting resin in the pad material does not occur.

## 4. Conclusions

This study investigated the squeal noise characteristics of the in-plane mode of the disc of a disc brake system according to the temperature of the brake pad. The instability before and after applying thermal deformation of the pad was analyzed using finite element analysis. The instability mode generated from the in-plane mode of the disc changed into an out-of-plane mode by the *z*-axis deformation of the pad. In addition, the analysis of the change in the magnitude of the real part of the in-plane mode according to the temperature revealed that near 80 °C was the most sensitive region for instability. Finally, squeal noise characteristics according to the pad geometry, temperature, pressure, and rotation speed were analyzed using a brake dynamometer to verify the reliability of the finite element analysis. The main frequency of squeal noise was approximately 10,000 Hz, which was derived to result from the instability of the in-plane mode derived from the analysis. In particular, the frequency of squeal noise was highest near 100 °C, whereas squeal noise disappeared after 250 °C. This tendency was largely consistent with the results of the finite element analysis, thereby verifying the reliability of the analysis.

In future research, we plan to develop a refined squeal model that more accurately reflects thermal effects by integrating mathematical modeling at the contact interface with machine learning techniques. This approach aims to establish a systematic methodology for the precise prediction of brake squeal behavior.

## Figures and Tables

**Table 1:** Material properties.

Components	Density [tonne/mm^3^]	Young’s Modulus [MPa]	Poisson’s Ratio	Thermal Expansion [mm/mm °C]
Pad	3.16200 × 10^−9^	14,500	0.27	1.04800 × 10^−5^
Backplate	7.51010 × 10^−9^	220,600	0.37	1.30000 × 10^−5^
Disc	6.94650 × 10^−9^	124,731	0.20	1.30000 × 10^−5^

**Table 2:** Modal analysis results of the backplate.

Mode	Mode Shape	Test (Hz)	FEM (Hz)	Error (%)
1	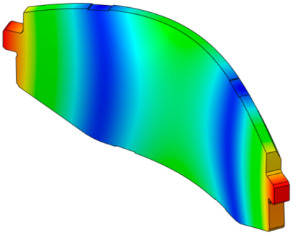	1676.0	1675.4	0.0
2	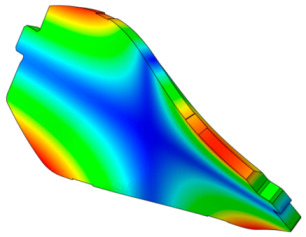	2626.7	2630.0	0.1
3	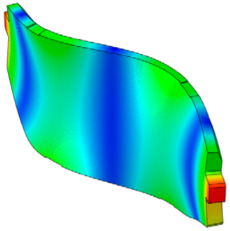	4258.9	4261.4	4.7

**Table 3:** Modal analysis results of the pad assembly.

Mode	Mode Shape	Test (Hz)	FEM (Hz)	Error (%)
1	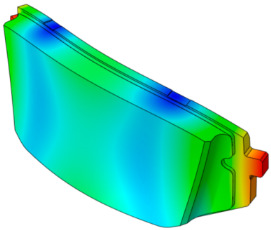	2890.0	2883.7	0.2
2	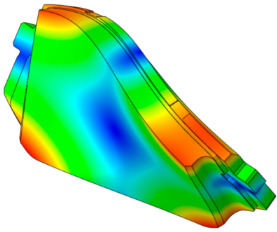	3935.3	3941.4	0.2
3	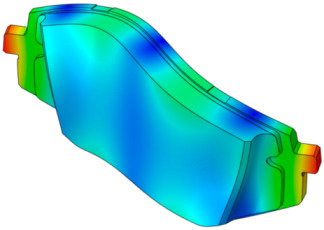	5958.7	6227.3	4.5

**Table 4:** Modal analysis results of the disc.

Mode	Mode Shape	Test (Hz)	FEM (Hz)	Error (%)
1	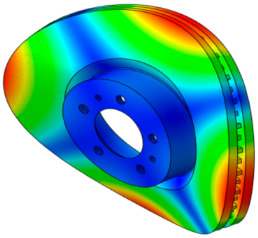	860.0	860.0	0.0
2	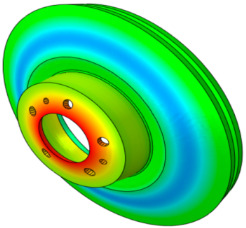	1582.0	1586.8	0.3
3	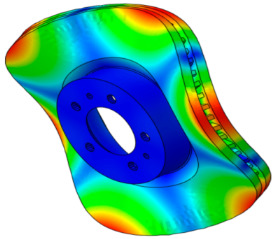	1974.0	1992.5	0.9

**Table 5:** Warm drag test condition by SAE J252.

Module	Section Description	Initial Speed [km/h]	Initial Temperature [°C]	Pressure [bar]
1	Break in	80	100	300
2	Burnish	80	100	a*
3	Friction characteristic	80	100	30
4	Drag	3, 10	b*	0, 30, 5, 25, 10, 20, 15
5	Warm-up	50	100, 150	30, 5, 25, 10, 20, 15
6	Drag (back/forward)	−3, 3	c*	0, 20, 5, 15, 10
7	Deceleration stop	50	d*	30, 5, 25, 10, 20, 15
8	Friction characteristic	80	100	30

a*: 15, 30, 15, 18, 22, 38, 15, 26, 18, 34, 15, 26, 15, 22, 30, 46, 26, 51, 22, 18, 42, 15, 18, 46, 26, 15, 34, 22, 18, 30, 18, 38. b*: 50, 75, 100, 125, 150, 175, 200, 225, 250, 300, 250, 225, 200, 175, 150, 125, 100, 75, 50. c*: 150, 125, 100, 75, 50. d*: 50, 100, 150, 200, 250, 200, 150, 100, 50.

## Data Availability

The raw data supporting the conclusions of this article will be made available by the authors on request.
